# Effect of antibiotic use on the efficacy of nivolumab in the treatment of advanced/metastatic non-small cell lung cancer: A meta-analysis

**DOI:** 10.1515/med-2021-0272

**Published:** 2021-05-11

**Authors:** Geng-wei Huo, Ran Zuo, Ying Song, Wei-dong Chen, Wen-ming Chen, Dao-qun Chong, Hong-mei Zhang, Sha-sha Jia, Peng Chen

**Affiliations:** Department of Thoracic Oncology, Tianjin Medical University Cancer Institute and Hospital, National Clinical Research Center for Cancer, Key Laboratory of Cancer Prevention and Therapy, Tianjin, China; Lung Cancer Diagnosis and Treatment Center, Tianjin’s Clinical Research Center for Cancer, Tianjin 300060, China; Department of Oncology, Jining No. 1 People’s Hospital, Jining 272000, Shandong, China; Department of Pharmacy, Jining No. 1 people’s Hospital, Jining 272000, Shandong, China; Department of Thoracic Oncology, Tianjin Medical University Cancer Institute and Hospital, National Clinical Research Center for Cancer, Tianjin, China

**Keywords:** non-small cell lung cancer, nivolumab, antibiotics, survival, meta-analysis

## Abstract

This study evaluates the impact of the use of antibiotics on the effectiveness of nivolumab in the treatment of advanced/metastatic non-small cell lung cancer (NSCLC). A literature search was conducted in various electronic databases to identify studies, which evaluated the impact of antibiotic use on the survival of patients with advanced/metastatic NSCLC who have been treated with nivolumab. Six studies, comprising a total of 787 patients with 37.2% females and of age range 30–90 years, were included in the study. A lack of smoking history was reported in 14.4% of the patients. A meta-analysis was conducted in 678 and 713 patients for PFS and OS, respectively. The pooled HR was 1.95 (95% CI: 1.13–3.37, *P* = 0.016) for PFS and 2.70 (95% CI: 1.81–4.02, *P* < 0.001) for OS. Among patients exposed to antibiotics, the median PFS and OS were reduced by 1.6 months (95% CI: 1.5–1.7) and 8.8 months (95% CI: 8.5–9.1), respectively. Our study indicates that, among patients with advanced/metastatic NSCLC, the use of antibiotics with nivolumab led to a decrease in the median OS by more than 8 months. Studying the mechanism of the effect of antibiotics on the efficacy of nivolumab in patients with NSCLC should also be prioritized.

## 1 Introduction

Lung cancer is a leading cause of cancer-related mortality with a global estimate of more than 1.8 million deaths occurring each year [1]. Depending on the histological features, lung cancers are classified into either small-cell lung cancer (15–20%) or non-small-cell lung cancer (80–85%) [2]. There has been a tremendous progress in the treatment of non-small cell lung cancer (NSCLC) in the past 15 years. The first-line treatment regimens for advanced NSCLC are based on driver oncogenes. With targeted therapies, the median survival can improve from 18.6 to 30.5 months in the case of tyrosine kinase inhibitors targeting epidermal growth factor receptor (EGFR) mutations and 4 years for treatment targeting anaplastic lymphoma kinase (ALK) alterations. However, most patients with NSCLC do not harbor these oncogenic drivers, limiting their treatment options to cytotoxic chemotherapy with or without bevacizumab. This treatment regimen is associated with a median survival of approximately 12 months and has a poor adverse event profile [3,4].

Paucity in the development of traditional chemotherapeutic drugs and resistance of some forms of cancers to available therapies has spurred the emergence of immunotherapy targeting programmed cell death protein 1 (PD-1), programmed cell death 1 ligand 1 (PDL-1), and cytotoxic T-lymphocyte-associated protein 4 (CTLA-4) for the treatment of NSCLC [5].

Nivolumab is the first among immune checkpoint inhibitors approved for treating lung cancer. This approved use stemmed from promising results of CheckMate017 and CheckMate057 studies involving patients with advanced squamous NSCLC and advanced nonsquamous NSCLC, respectively. In both cases, nivolumab demonstrated better progression-free survival and overall survival with minor side effects compared to docetaxel, which was associated with a long-term survival, i.e., 4-year overall survival was 14% (95% CI: 11–18) in patients treated with nivolumab compared with 5% (95% CI: 3–7) in patients treated with docetaxel [6,7,8]. Notably, the efficacy of immune checkpoint inhibitors in the treatment of NSCLC patients varies, with most patients reported to develop acquired resistance [9]. The phase III CheckMate 026 trial tested the efficacy of nivolumab compared to the standard first-line chemotherapy in 423 patients with ≥5% PD-L1-positive advanced NSCLC. No benefit was seen with nivolumab compared to chemotherapy in terms of the primary endpoints PFS, OS, or RR [10]. Improving the efficacy of immune checkpoint inhibitors in the treatment of patients with NSCLC is a subject of clinical importance.

Patients with NSCLC often present with generally poor health and weakened immunity. As a result, the incidence of infection among these patients, and therefore the probability of using antibiotics, is relatively high [11]. Prior exposure to antibiotics or their concurrent use with immune checkpoint inhibitors can affect treatment efficacy among patients with NSCLC. Similar observations have been reported with the use of antibiotics after treatment using these agents [12,13,14]. Discordant findings on the effects of antibiotics on therapy using immune checkpoint inhibitors such as nivolumab motivate further studies to better understand this phenomenon. The aim of this study is to evaluate the response and survival rates of patients with advanced NSCLC who were treated using nivolumab with or without antibiotics.

## 2 Methods

### 2.1 Inclusion and exclusion criteria

To be included in the meta-analysis, a study had to (a) compare the efficacy of nivolumab when used alone versus when antibiotics had been used before, concurrently, or after the use of nivolumab in the treatment of NSCLC and (b) report the efficacy indices including progression-free survival (PFS) or overall survival (OS). Studies were excluded if (a) they reported outcomes that were influenced by other drugs besides antibiotics; (b) they were concerned only with pharmacokinetic or pharmacodynamic investigations; (c) they involved only *in vitro*, molecular, or experimental investigations; and (d) they provided only qualitative information.

### 2.2 Literature search

Several electronic databases including Google Scholar, PubMed, Science Direct, and proceedings of major oncology conferences were searched by using specific keywords and medical subject headings. Primarily, the search term “nivolumab antibiotics NSCLC” was used followed by several other extensions including the words penicillin, quinolone, response, survival, tumor, node, metastasis, and TNM. The scope of the search encompassed research articles published in English before October 2020. In addition, the bibliographies of important related papers were also screened.

### 2.3 Quality assessment

The quality assessment of the studies included in the meta-analysis was performed using the New Castle–Ottawa Scale for the Quality Assessment of Cohort Studies [15]. Random sequence generation (selection bias), allocation concealment (selection bias), blinding of participants and personnel (performance bias), blinding of outcome assessment (detection bias), incomplete outcome data (attrition bias), and selective reporting (reporting bias) were all evaluated independently by two authors.

### 2.4 Data and analyses

Data were extracted independently by two authors who reviewed and screened all eligible studies for content according to the inclusion criteria described previously. Information on the baseline demographics, clinical, oncological, and genetical data were recorded. Other data included the study design, methodology, the analysis performed, and the outcome noted from each study.

If both univariate and multivariate analyses were performed, we adopted the results obtained from multivariate analyses. For two studies [16,17] that lacked HR for PFS values, these parameters were estimated from the Kaplan–Meier curves using the spreadsheet attached to the publication. The calculations were repeated twice and independently to ensure consistency of the results [18]. The weighted average of median PFS and OS reported for patients exposed and those not exposed to antibiotics was also computed using the weight attributed in the meta-analysis.

The survival reported by individual studies was pooled under the random-effects model to achieve an overall effect size of each endpoint as an inverse variance weighted average of the individual study effect sizes. Statistical heterogeneity was estimated using *I* ^2^ index. The Egger linear regression test and Begg rank correlation methods were applied to determine publication bias. All statistical analyses were performed using Stata11.0 software (Stata A Corp, College Station, Texas, USA), and *p* < 0.05 was considered statistically significant.

## 3 Results

### 3.1 Literature search results

**Figure 1:**
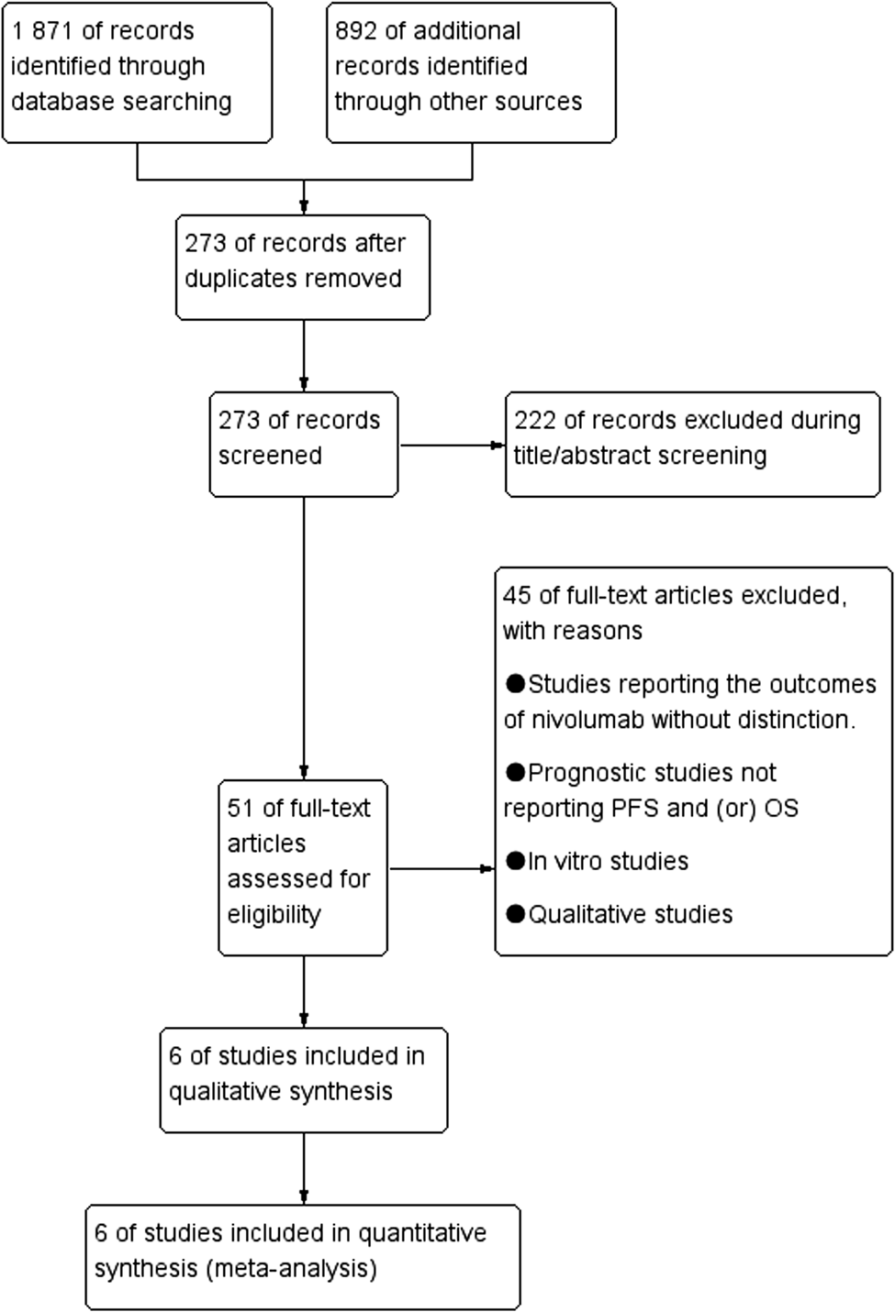
Flow diagram of the included studies.

Applying the research search strategy identified 2,763 potentially relevant records from databases and conferences. The selection process and reasons for the exclusion of ineligible studies are presented in Figure 1. A total of 2,757 studies were excluded after screening the abstract and the full text. Thus, six studies, all of which were retrospective, fulfilled the eligibility criteria and were included in the meta-analysis [16,17,19,20,21,22].

### 3.2 Characteristics of the selected studies

The social and clinical characteristics of the patients in the studies included in the meta-analysis are presented in Tables 1 and 2. All patients had locally advanced unresectable recurrent or metastatic NSCLC. Some 787 cases were treated using nivolumab. Of this, 205 cases were treated with antibiotics before, concurrently, or after treatment with nivolumab, while 582 cases were not treated with antibiotics. The age range of the patients in the studies was 30–90 years, and there were fewer females (37.2%). The minority of the patients (14.4%) had never smoked before. The studies had an acceptable degree of quality as summarized in Table 3.

**Table 1:** Basic characteristics of included studies (*n*)

Reference	Time window of ATB exposure (days)		Patients (ATB +/ATB−)	mPFS (months) (ATB+ vs ATB−)	mOS (months) (ATB+ vs ATB−)	HR for PFS (95% CI)	HR for OS (95% CI)	Quality
Do et al.	−30	30 after the last dose	87/22	NA	5.4 vs 17.2	NA	3.44 (1.72–6.67)	6
Hakozaki et al.	−30	0	13/77	1.2 vs 4.4	8.8 vs >15	3.55 (1.69–7.47)	2.02 (0.70–5.83)	7
Kaderbhai et al.	−90	CO	15/59	3.8 vs 2.3	NA	1.09 (0.57–2.10)	NA	7
Ouaknine Krief et al.	−60	30	30/42	2.8 vs 3.3	5.1 vs 13.4	1.6 (0.6–2.2)	2.2 (1.1–4.8)	7
Schett et al.	−60	0	33/185	1.4 vs 5.5	1.8 vs 15.4	3.86 (2.28–6.53)	4.29 (2.48–7.39)	7
Svaton et al.	−30	30	27/197	4.4 vs 6.0	12.8 vs 13.1	1.182 (0.642–2.178)	1.513 (0.717–3.193)	7

Abbreviations: CO – concurrently; NA – not available.

**Table 2:** Important characteristics of the included studies

Reference	Year	Design	Country	Ethnicity	*N*	Median age (years)	Females (%)	Never smokers (%)	PFS analysis type	OS analysis type	ECOG PS (%)			Stage (%)		
											0	1	2	Ⅲ	Ⅳ	Recurrent
Do et al.	2018	R	United States of America	C	109	NA	NA	NA	NA	U	NA	NA	NA	NA	NA	NA
Hakozaki et al.	2019	R	Japan	A	90	67 ± 8.5	36.7	NA	U	M	0–1:71.1		14.4	0	55.6	44.4
Kaderbhai et al.	2017	R	France	C	74	67 ± 5.7	18.9	12.2	U	NA	40.5	55.4	4.1	NA	NA	NA
Ouaknine Krief et al.	2019	R	France	C	72	68.4 ± 8	38.0	13	M	M	0–1:63.4		36.6	8.3	91.7	0
Schett et al.	2019	R	Switzerland	C	218	64 ± 10	40.0	10.6	U	U	26.1	43.1	11.0	11.5	88.5	0
Svaton et al.	2020	R	Czech	C	224	67	40.6	19.2	M	M	25.0	73.2	21.8	10.7	89.3	0

Abbreviations: A – Asian; C – Caucasian; M – multivariate analysis; NA – not available; R – retrospective; U – Univariate analysis.

**Table 3:** Quality assessment of the included study with New Castle–Ottawa quality assessment scale

Study	Representativeness of exposed cohort	Selection of nonexposed cohort	Ascertainment of exposure	Demonstration that outcome of interest was not present at start of study	Comparability of cohorts on the basis of the design or analysis	Assessment of outcome	Was follow-up long enough for outcomes to occur	Adequacy of follow-up completion of cohorts
Do et al.	*	*	*	*		*	*	
Hakozaki et al.	*	*	*	*		*	*	*
Kaderbhai et al.	*	*	*	*		*	*	*
Ouaknine Krief et al.	*	*	*	*		*	*	*
Schett et al.	*	*	*	*		*	*	*
Svaton et al.	*	*	*	*		*	*	*

* Adequacy of criteria and its absence represents inadequacy.

### 3.3 Effects of antibiotics on PFS

**Figure 2:**
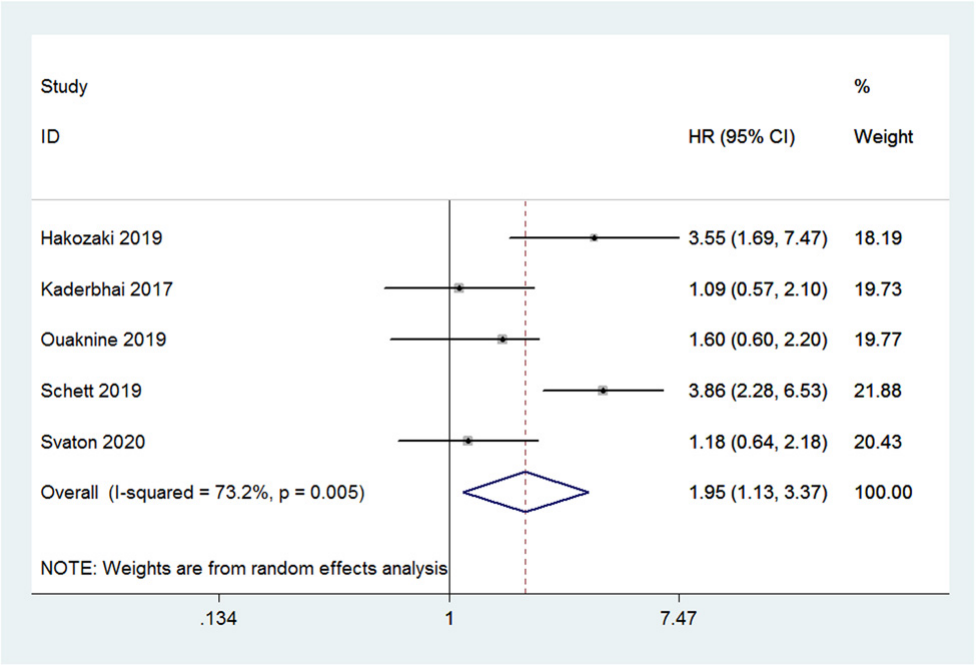
Forest plot of the pooled PFS based on exposure to antibiotics.

Five of the included studies reported PFS results. There was a significant improvement in the PFS among NSCLC patients treated with nivolumab and who were not exposed to antibiotics compared to those in whom antibiotics were used (HR: 1.95, 95% CI: 1.13–3.37, *P* = 0.016; Figure 2). There was high, and significantly different, heterogeneity in the PFS (*I* ^2^ = 73.2%; *P* = 0.005). Applying the same weighting of studies, the median PFS among patients exposed to antibiotics reduced by an average of 1.6 months (95% CI: 1.5–1.7).

### 3.4 Effects of antibiotics on OS

**Figure 3:**
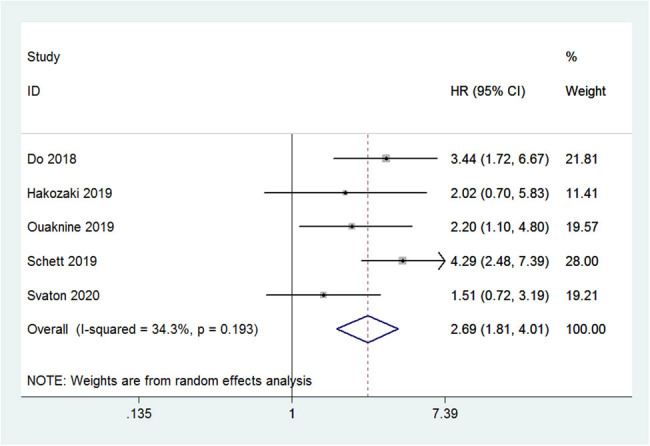
Forest plot of the pooled OS based on exposure to antibiotics.

In the five studies that reported on the OS, patients with NSCLC, who received treatment with nivolumab without antibiotics, experienced superior OS when compared to those in whom antibiotics were used (HR: 2.70; 95% CI: 1.81–4.02; *P* < 0.001; Figure 3). A low heterogeneity, although not statistically significantly different, was observed for the OS (*I* ^2^ = 34.3%; *P* = 0.193). When the same weighting of studies was used, the median OS among patients who were exposed to antibiotics was reduced by an average of 8.8 months (95% CI: 8.5–9.1).

### 3.5 Publication bias and sensitivity analysis

**Figure 4:**
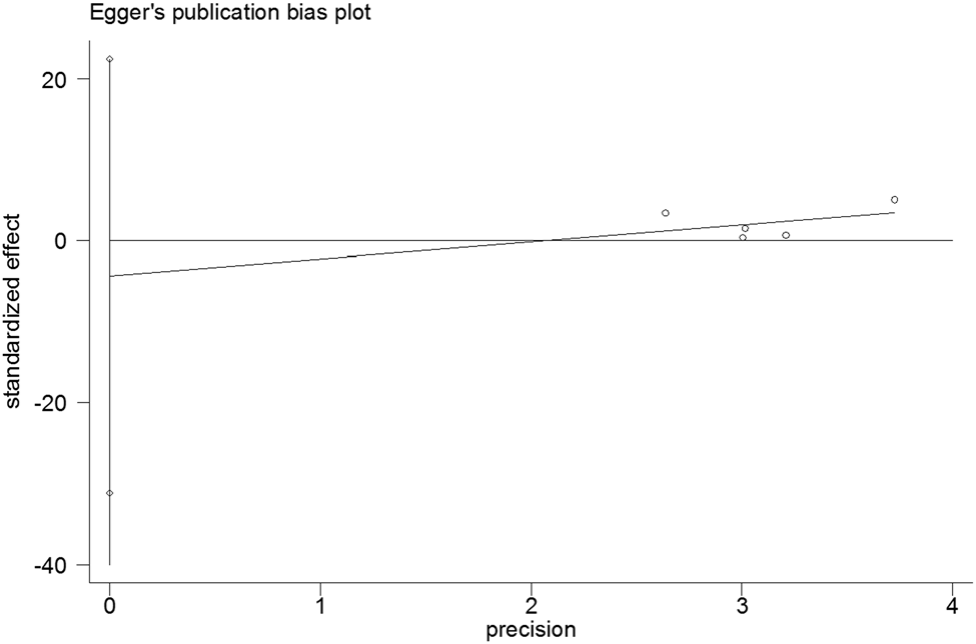
Egger linear regression test for PFS in patients exposed to antibiotics versus those not exposed to antibiotics at the initiation of nivolumab treatment.

**Figure 5:**
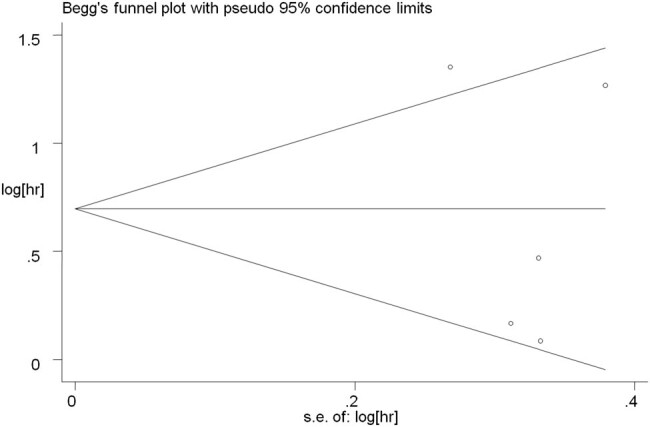
Begg’s funnel plots for evaluating publication bias of PFS.

**Figure 6:**
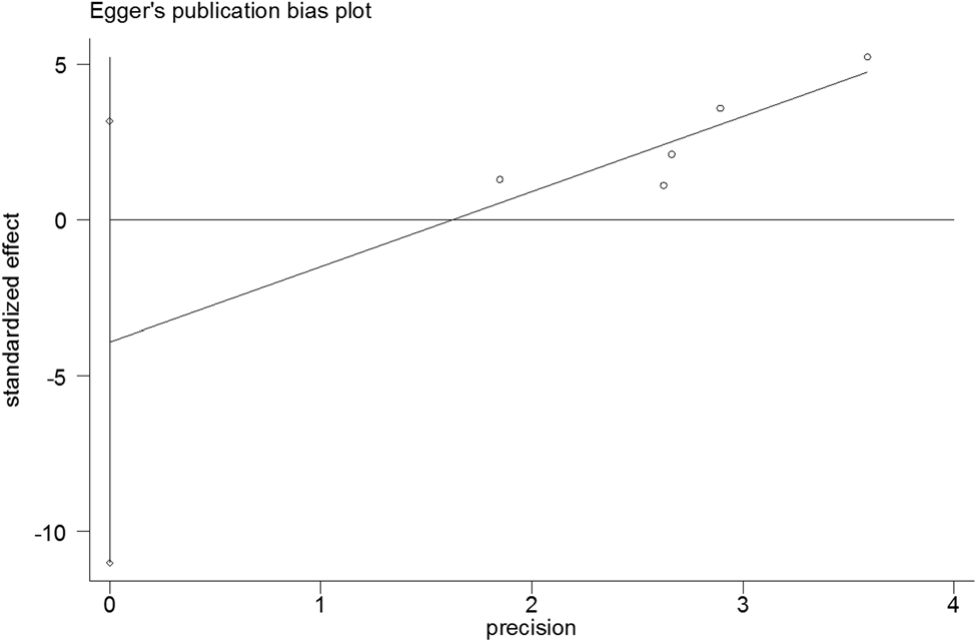
Egger linear regression test for OS in patients exposed to antibiotics versus those not exposed to antibiotics at the initiation of nivolumab treatment.

**Figure 7:**
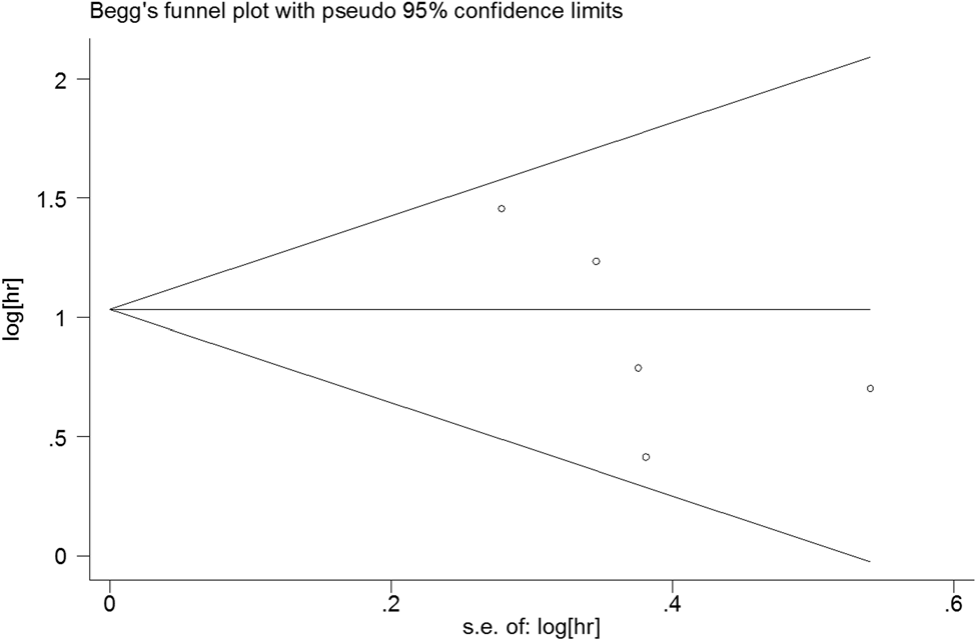
Begg’s funnel plots for evaluating publication bias of OS.

**Figure 8:**
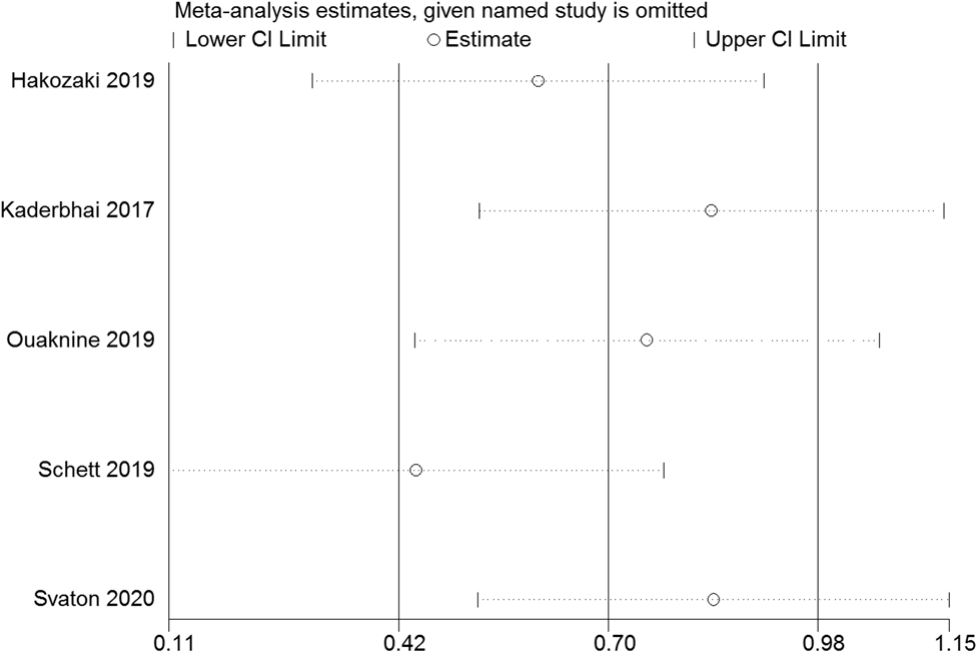
Sensitivity analysis of the studies assessing PFS.

**Figure 9:**
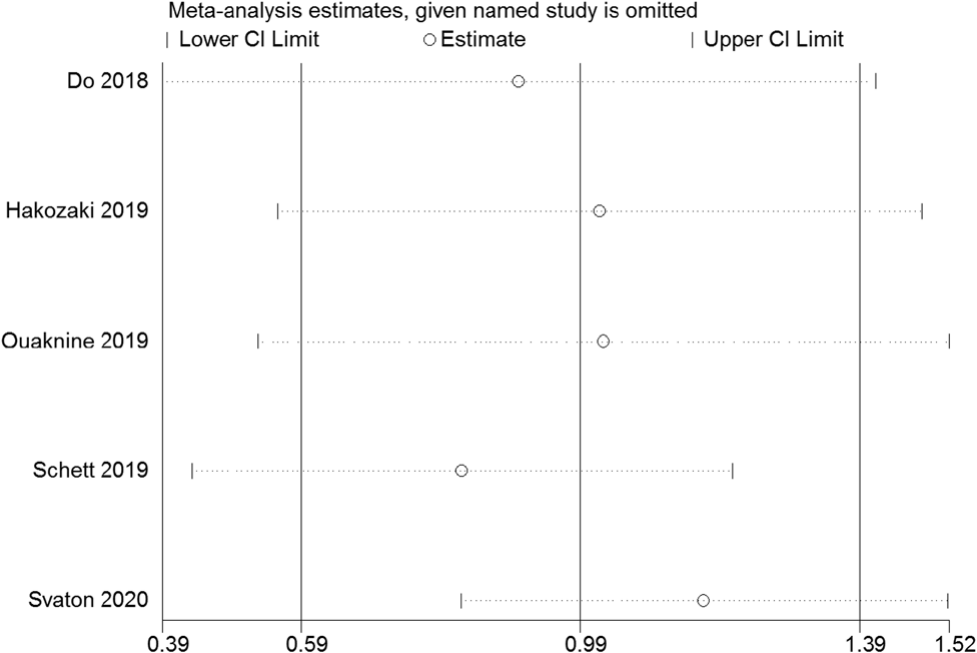
Sensitivity analysis of the studies assessing OS.

The Egger linear regression test and Begg rank correlation method verified that there was no obvious publication bias in the PFS (Egger’s test: *t* = −0.52, *P* = 0.639; Begg’ test: *z* = 0.24, *P* = 0.806; Figures 4 and 5) and OS (Egger’s test: *t* = −1.76, *P* = 0.177; Begg’ test: *z* = 1.22, *P* = 0.221; Figures 6 and 7). Furthermore, sensitivity analysis indicated that no point estimate of the omitted individual dataset lay outside the 95% CI of the combined analysis (Figures 8 and 9). Taken together, these outcomes indicate that the results of this meta-analysis were reliable.

## 4 Discussion

Our study found that the prior, concurrent, or subsequent use of antibiotics among patients with advanced NSCLC being treated with nivolumab correlates with a lower PFS and OS. To the best of our knowledge, this is among the first meta-analysis studies to explore the impact of antibiotics use on the survival of patients suffering from advanced NSCLC and on the treatment using nivolumab.

In recent years, the role of intestinal microbiota in the treatment of immune checkpoint inhibitors has attracted the attention of researchers. The composition of intestinal microbiota in the general population varies greatly depending on, among other factors, race, age, antibiotic use, disease, and diet [23]. The use of antibiotics can extensively affect the intestinal microflora in a relatively short period of time [24] and lead to an imbalance in a person’s intestinal flora composition [25]. This is a potential outcome that may affect the effectiveness of immune checkpoint inhibitors.

In a study involving tumor-bearing mice raised with broad-spectrum antibiotics in a sterile environment for 14 days, the antitumor effect of PD-1 inhibitors was significantly worse than those of mice raised in a normal environment. Fecal microbiota transplantation from NSCLC patients who responded to PD-1 inhibitors into antibiotic-treated mice restored the antitumor effects of the PD-1 inhibitors [26]. A study among Chinese patients with advanced NSCLC revealed a strong correlation between gut microbiome diversity and the response to anti-PD-1 immunotherapy. Patients with a high diversity of gut microbiome exhibit enhanced memory T-cell and natural killer cell signatures in the periphery. This appears advantageous in giving them longer PFS (mPFS 209 days vs 52 days, *P* = 0.005) [27].

It is often inevitable, in the course of immunotherapy, for patients to require to use antibiotics due to infections. Therefore, it becomes important to establish how best to minimize the potential negative impact of antibiotics when treating patients using nivolumab.

In another study, the oral administration of bifidobacteria in tumor-bearing mice significantly enhanced the antitumor effect of PDL-1 inhibitors. This observation is ascribed to improved function of dendritic cells with increased infiltration of CD8+ tumor-specific T cells into the tumor microenvironment, leading to the production of a large amount of IFN-γ [28]. Based on this reasoning, the oral administration of bifidobacteria may reduce the effect that antibiotics can have on the efficacy of nivolumab. However, this hypothesis needs to be further verified through clinical trials.

Our study, despite providing interesting insights, has some limitations. For instance, patients who use antibiotics are, usually, in a poorer state of health compared to those who are not on antibiotics. Therefore, it is not always possible to accurately capture the information on the disease progression profile. Variations in the timing and duration of antibiotic use, cancer staging of the patient, patient pretreatment conditions, and HR adjustment factors cause heterogeneity in the PFS data among studies. Since there are currently no large-scale randomized controlled clinical trials that study the impact of antibiotics on the efficacy of nivolumab among patients with NSCLC, the influence of multiple confounding factors cannot be ruled out from the available retrospective studies.

In spite of the stated limitations, it is clear that the survival of patients with NSCLC being managed with nivolumab is compromised by the use of antibiotics. Therefore it is important that, whenever immune checkpoint inhibitors are used in such patients, antibiotics should be used cautiously, weighing the risks and benefits. Appropriate timing, duration, and choice of antibiotics, as well as the restoration of intestinal flora composition after antibiotic exposure, are expected to minimize the impact of antibiotics on the efficacy of nivolumab.

In conclusion, our study shows that the use of antibiotics among patients suffering from NSCLC is associated with a decrease in survival. While discordant results invite the challenge for future larger prospective studies to verify the results, clinicians are encouraged to adopt strategies that optimize antibiotic use among such patients. We recommend further research on the molecular mechanisms of the effect of antibiotics use on the efficacy of nivolumab in the treatment of patients with advanced NSCLC.
